# Influence of the usual motivation for dental attendance on dental status and oral health-related quality of life

**DOI:** 10.4317/medoral.19366

**Published:** 2013-10-13

**Authors:** Javier Montero, Alberto Albaladejo, José I. Zalba

**Affiliations:** 1 DDS. PhD in Dentistry. Graduate in Odontology. Tenured Lecturer of Faculty of Medicine. University of Salamanca. Campus Miguel de Unamuno. Salamanca, Spain; 2 DDS. PhD in Dentistry. Graduate in Odontology. Private Practice on Preventive and Minimally Invasive Dentistry. Pamplona, Spain

## Abstract

Objectives: To evaluate the influence of dental visiting patterns on the dental status and Oral Health-related Qua-lity of Life (OHQoL) of patients visiting the University Clinic of Salamanca (Spain). Study Design: This cross-sectional study consisted of a clinical oral examination and a questionnaire-based interview in a consecutive sample of patients seeking a dental examination. Patients were classified as problem-based dental attendees (PB) and regular dental attendees (RB). Clinical and OHQoL (OHIP-14 & OIDP) data were compared between groups. Pair-wise comparisons were performed and a Logistic Regression Model was fitted for predicting the Odds Ratio (OR) of being a PB patient. Results: The sample was composed of 255 patients aged 18 to 87 years (mean age: 63.1 ± 12.7; women: 51.8%). The PB patients had a poorer dental status (i.e. caries, periodontal and prosthetic needs), brushed their teeth less,and were significantly more impaired in their OHQoL according to both instruments. The logistic regression coefficients demonstrated that on average the OR of being a PB patient was high in this dental patient sample, but this OR increased significantly if the patient was a male (OR= 1.1-5.0) or referred pain-related impacts according to the OHIP and, additionally, the OR decreased significantly as a function of the number of healthy fillings and the number of sextants coded as CPI=0. Conclusions: Regular dental check-ups are associated with better dental status and a better OHQoL after controlling for potentially related confounding factors.

** Key words:**Dental attendance, oral health-related quality of life.

## Introduction

A key behavioural indicator that has been used since the first Adult Dental Health Survey of England and Wales in 1968 is whether people say they go to a dentist for a regular dental check-up or only when dental problems arise (1). During the last three decades, several studies have reported that regular dental attendees are less likely to suffer from the acute symptoms of dental disease, and that fewer tend to require emergency treatment than problem-based dental attendees (1-3). Adults who do not attend dental check-ups are more likely to have a poorer dental status and worse subjective oral health than people who usually attend dental check-ups (3-6). It also has been found that the pattern of dental visiting (regular versus irregular) is clearly related to age and gender but is also partially modulated by some psychological (anxiety, health beliefs...), educational and financial factors (1,4,7). Fear, anxiety and costs have been shown to be the major causes for not attending dentists regularly (1,4).

In most Western countries, it has been estimated that about half of the adult population are routine attendees (8), the rates being lower among men and in certain social, ethnic, or age groups, and higher in Scandinavia (9) and Britain (1). In Spain, the expressed motivation for dental attendance has not been reported previously, but according to the last National Health Survey in Spain performed in 2011-2012 (data available from the National Institute of Statistics from www.ine.es) about 50 to 55% of people aged from 15 to 64 years reported that their last visit to the dentist had taken place about one year previously or longer. This percentage is gradually becoming higher among the elderly (aged ≥65 yrs) ranging from 65% to 80%. The type of treatment received at the last visit was mostly conservative among teenagers and adults (check-up, tooth cleaning or fillings) but, by contrast, the last treatment received was more invasive for the elderly (exodoncy or prostheses).

Oral diseases are not usually fatal, but can affect the ability to eat, speak and socialise… Thus, there is currently anemerging interest in how oral health and oral behaviour affects the quality of life. Oral health-related quality of life (OHQoL) has been defined as the extent to which oral disorders or conditions affect functioning and psychosocial well-being (10). For OHQoL assessment, a large variety of instruments (questionnaire and scales) has been developed over the past three decades (11), generally being applied as descriptive measures in cross-sectional studies. Recent epidemiological studies have found that problem-oriented attendees have a poorer dental status and a lower oral-health-related quality of life (5,12-14).

Accordingly, understanding the impact of the motivation to receive dental attendance on the oral health status and quality of life may hold the key to determining and quantifying the importance of this behavioural pattern.This study aims to investigate the impact of dental visiting patterns (problem-based versus regularly) on dental status and OHQoL among dental patients attending the University Dental Clinic of Salamanca (Spain).

## Material and Methods

The study was approved by the Bioethics Committee of the University of Salamanca and all participants gave specific (written) informed consent. A consecutive sample of patients seeking dental examination was recruited during 2010 and 2011. Patients were classified according to the underlying motivation for dental attendance, dichotomizing the sample into those reporting problem-based dental visits (PB) and those reporting regular check-ups (RB), from annually to every two years. Patients were asked: “In general do you attend check-ups regularly or only when you have some trouble with your teeth? This categorization has been used previously (6).

The study consisted of a clinical oral examination and a questionnaire-based interview. All patients were examined clinically according to WHO guidelines by a trained examiner (JM) and data on caries and periodontal status (CPI: Community Periodontal Index) were collected. Furthermore, we recorded the number of occlusal and aesthetic units by visual inspection. In the former case, we counted the natural or fixed-prosthesis-replaced occlusal pairs in the premolar and molar areas while the subjects maintained the maximum intercuspal position of the jaw stable. By contrast, the count of aesthetic units only took values between zero and six on recording the natural or fixed-prosthesis-replaced aesthetic pairs of teeth (between canines).

In addition, all patients were interviewed in a face-to-face interview performed by trained staff using two widely used OHQoL indicators i.e. the Spanish OHIP-14 (15) and the Spanish OIDP (16). The Spanish OHIP-14 is a questionnaire that evaluates the frequency of the appearance of impacts in 7 dimensions (pain, functional limitation, psychological discomfort, physical, psychological, and social incapacity and disability) using a Likert scale from 0 to 4 (0=never; 1= rarely; 2= occasionally; 3= fairly often, and 4= very frequently). The OIDP captures both the severity and the frequency of oral-related problems or difficulties in 8 dimensions (eating, speaking, hygiene, occupational, social, smiling, sleeping-relaxing, emotional). The severity of the impact on everyday life ranges from a very minor effect (coded as zero) to a very severe effect (coded as five).Both instruments have good psychometric properties and their strengths could be complementary in assessing the impact of oral health on quality of life (17).

The summary scores of both instruments, whichare proportional to the impact in theOHQoL, were calculated as follows. For OHIP, we calculated the number of items recorded as “occasional” or more frequently (“items with impact”). For the OIDP we summed the number of items recorded as having a fairly minor or more severe effect one very day life. Moreover, foreach domain we calculated the dimensional impact. For the OHIP, the dimensional impact was the mean of items with impact (two items per domain). For each of the eight domains of the OIDP we calculated the dimensional impact by multiplying the frequency and severity scores recorded.

Furthermore, global oral satisfaction was determined using a 0-10 Visual Analogue Scale (VAS) previously used among the reference population (16).

-Statistical analyses

Student’s ttests were used to compare quantitative variables among both groups. A Forward Stepwise logistic Regression Model was fitted to predict the Odds Ratio (OR) of being a problem-based patient (PB), using all the potential predictors revealed in the previous bivariate analysis. The Nagelkerke R2 was used to estimate the model fit. The analysis was run with the SPSS v19 (Statistical Package for Social Sciences; Chicago, IL) and graphics were obtained with STATA v12 (StataCorp LP; LakewayDrive, Tx,).

## Results

The study sample comprised 255 patients aged 18 to 87 years (mean age: 63.1 ± 12.7; women: 51.8%). 82% of the subjects reported that the usual motivation for dental attendance was problem-related (PB patients), the counterparts being those patients referring to regular attendance at check-ups (RB patients).

 Table 1 shows the different sociodemographic, behavioural and clinical profiles of both groups. The RB Patients were mostly women and were significantly younger than the PB patients. The RP patients brushed their teeth more frequently than their counterparts. 84.4% of the RB patients brushed their teeth at least twice a day, in contrast to the 61% of the PB patients. The social class distribution was similar in both groups, although we found a higher proportion of qualified workers (22.2% versus 13.3%) in the RB patients than in the PB patients. In addition, the proportion of unskilled manual workers was higher in the PB patients (31.4%) than in the RB patients (24.4%).

**Table 1:**
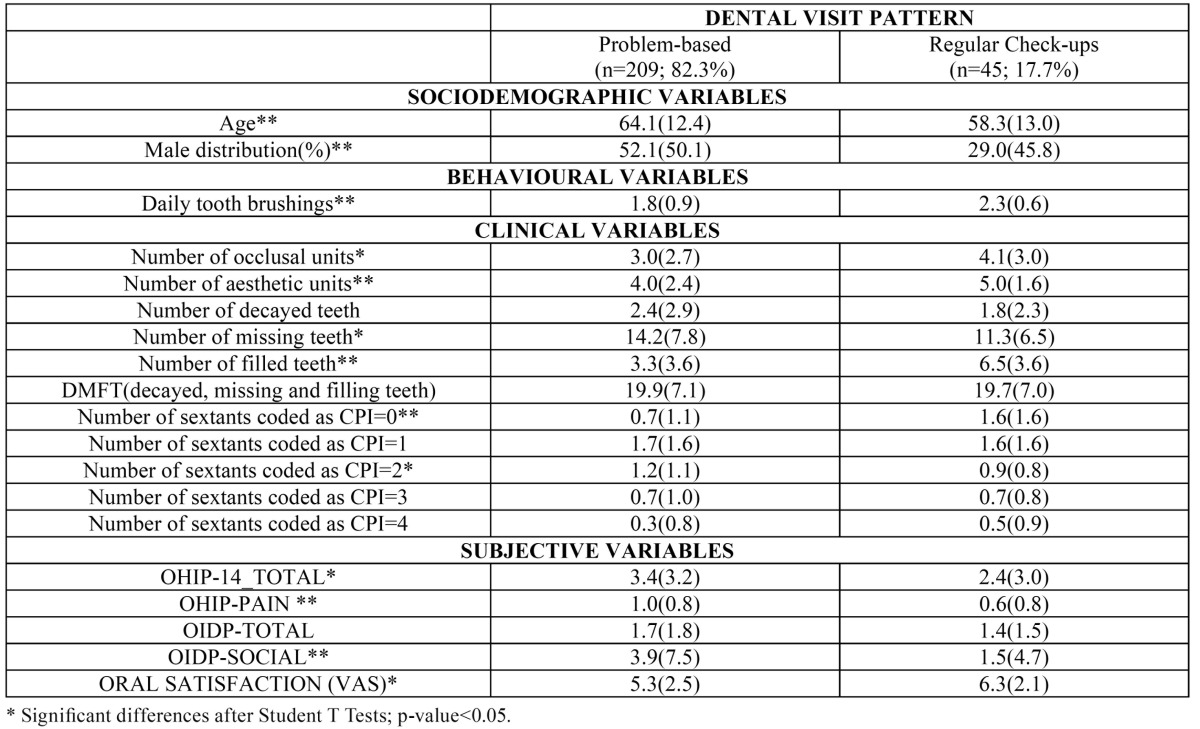
Sociodemographic, clinical and subjective variables among PB and RP patients. Inter-group comparisons by T-tests.

Clinically, the RB patients showed a significantly better dental and periodontal status, affording better oral well-being, as indicated by the mean total summary scores of OHIP, OIDP and the oral satisfaction scale. The average impact on the “pain” dimension, according to the OHIP, and ”social” dimension, according to the OIDP, was significantly lower. However, the DMFT and the number of sextants with moderate or severe periodontal pockets were similar in both groups. Nevertheless, the components of the DMFT index were significantly different, since the RB patients had significantly more filled teeth butfewer missing teeth.

Since all the pair-wise relationships depicted in  Table 1 could act as confounders or effect modifiers, a logistic regression including all these potentially related variables was performed for predicting the odds ratio of being a PB patient. The logistic regression coefficients ( Table 2) demonstrated that on average the OR of being a PB patient was high within this sample (OR=7.2) but this OR was increased significantly if the patient was a man (OR= 1.1-5.0) or referred to pain-related impacts according to the OHIP. By contrast the OR decreased significantly as a function of the number of healthy fillings and the number of sextants coded as CPI=0. The goodness of fit according to the Nagelkerke R2 was 0.27 and the proportion of the PB patients properly predicted by the model was 95.2% (globally, the model correctly classified subjects in 83.5% of the observations).

**Table 2:**
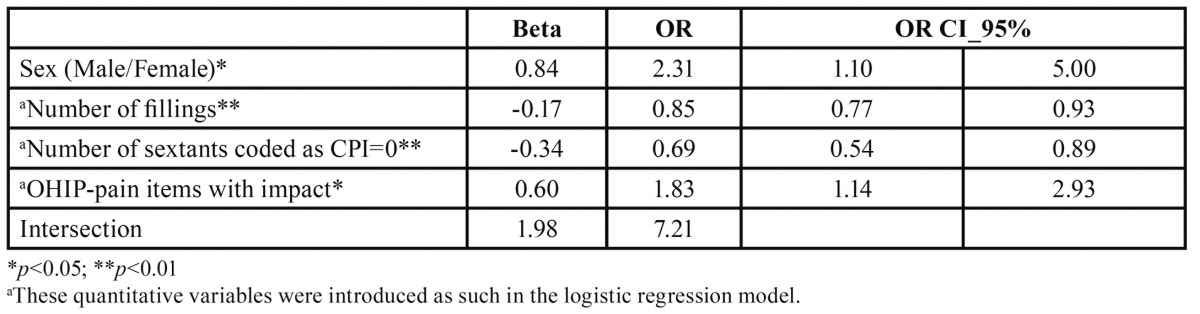
Prediction of the risk of being a PB patient according to thelogistic regression in which all potential predictors were included after a forward step-wise Wald selection method.

 Table 3 and  Table 4 show that the impact among the PB patients was higher with both instruments (OHIP and OIDP) and across all dimensions, the pain and social-related dimensions being those most clearly detected.  Table 3 and  Table 4 show that a large proportion of the RB patients did not report any impact on their OHQoL according to the OHIP and OIDP respectively.  Table 3 and  Table 4 show that the impact level within some OHIP and OIDP domains are significantly greater in PB-patients; i.e the“functional limitation” and “pain” according to the OHIP-domains ( Table 3), and “eating”, “speaking” and “social” dimensions according to the OIDP ( Table 4).

**Table 3:**
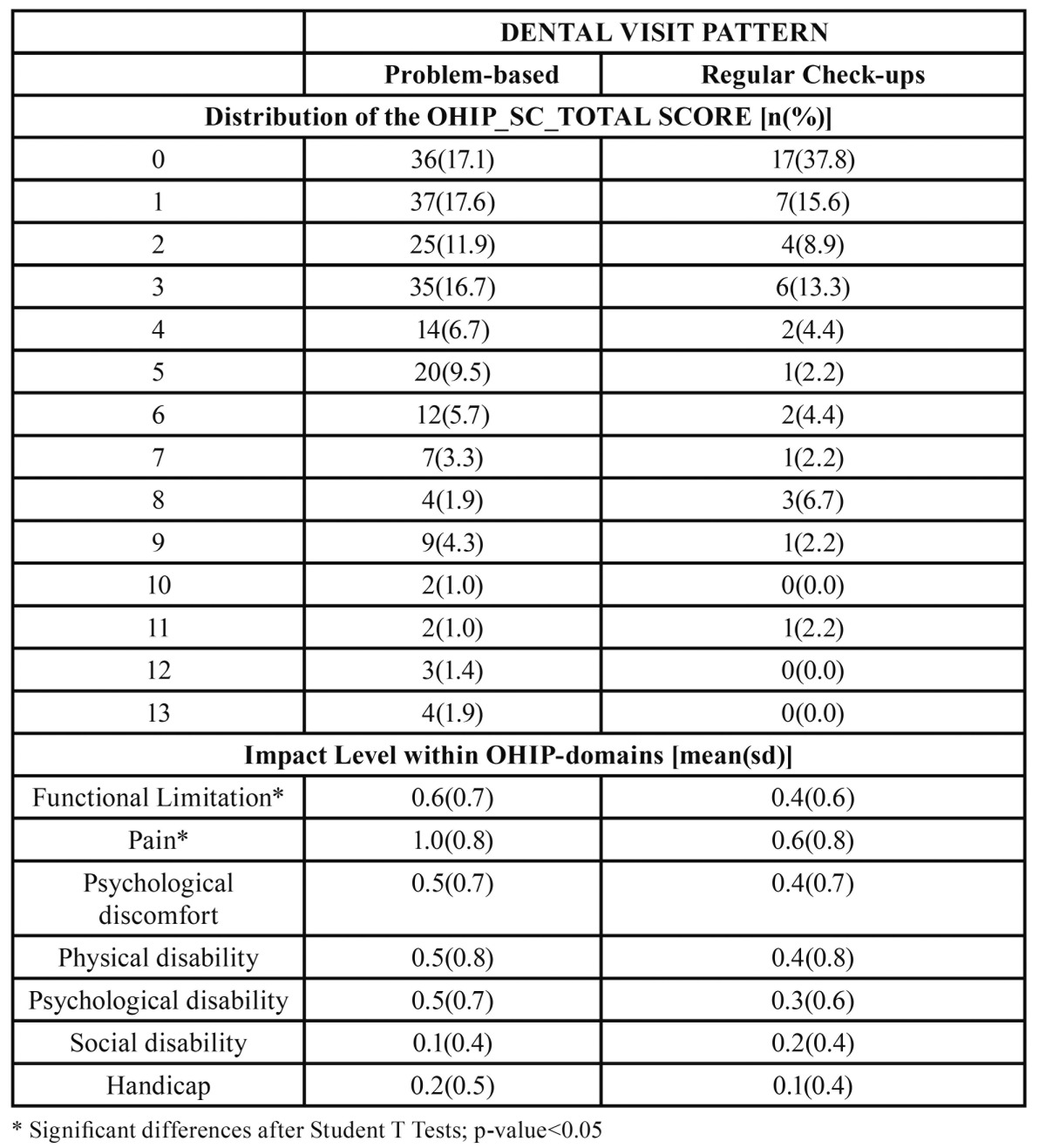
Distributions of the OHIP summary scores and their averaged dimensional impacts among problem-based and regular-based dental patients. Inter-group comparisons by T-tests.

**Table 4:**
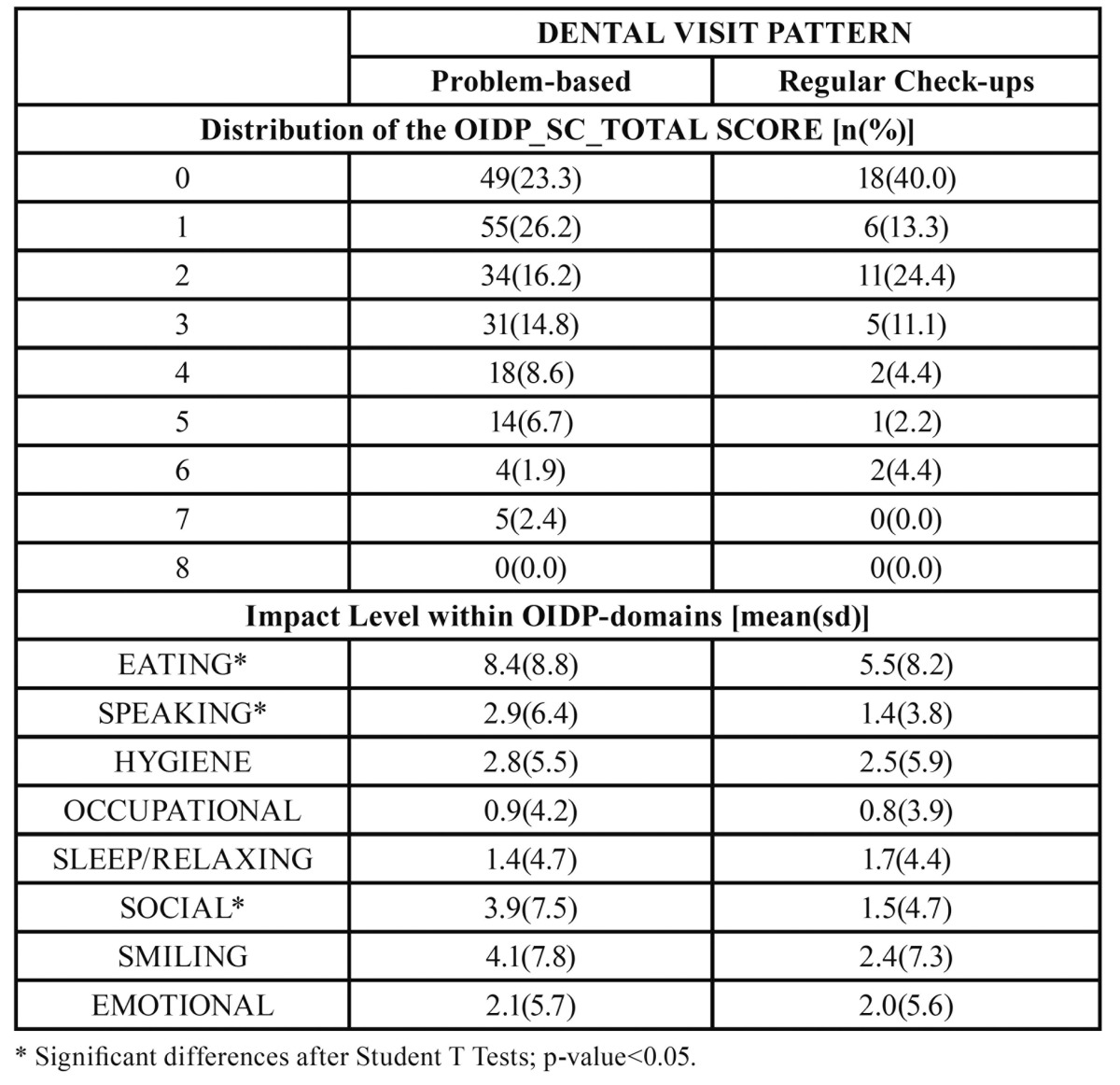
Distributions of the OIDP summary scores and their averaged dimensional impacts among problem-based and regular-based dental patients. Inter-group comparisons by T-tests.

## Discussion

This study focuses on assessing the impact of the dental visiting pattern (problem-based versus regularly) on the dental status and the OHQoL of dental patients attending the University Dental Clinic of Salamanca (Spain). The dental visiting pattern is only one part the recommendable oral health habits but it has not been properly evaluated in the Spanish population nor in dental patients.

Considering the weaknesses and strengths of the study, it must be acknowledged that this was not an epidemiological study and the results found are not applicable to the general Spanish population. In addition, since the recruitment sampling point was a University Dental Clinic in which dental treatment costs are clearly lower than in private dental clinics, the socio-demographic profile of this sample is clearly different (lower) from that of the general population. However, this could also be of interest, since both groups belonged to a comparable socio-demographic profile (which is a widely known confounding factor). Moreover the dental attendance data were self-reported, and we were unable to verify each participant’s dental attendance or the period in which this occurred, so it is possible that some of the subjects may have changed their pattern(e.g., from non-routine to routine or viceversa). This is the reason of questioning subjects thus: “*In general do you attend check-ups regularly or only when you have some trouble with your teeth*? As done by other authors (1,3).

Moreover, we did not record the reported underlying causes of this key pattern in spite of it being recognized that beliefs, attitudes, fears, economic costs, and dental education (1,18,19) may play an important role in this context. Future studies should address the underlying factors, being aware that the assessment of such a construct is arduous (20). The main strength of the study lies in its mix of standardized clinical and self-report outcome measures (OIDP, OHIP-14 and Oral Satisfaction) on a consecutive sample seeking dental assessment.

The findings of this study show that among the study sample people who attend dental consultations regularly are less common (17.7%) than the figures reported for British (19), or Swedish adults (9) or young adults from New Zealand (8), whose percentages of regular attendees are above 50%. This was expected since ours was a cross-sectional study performed on patients and not an epidemiological study carried out on the general population. Indeed, most of participants included in the present study were assumed to be searching for a cheaper way to treat their dental treatment needs, and dental costs have proved to be an important factor in the regularity of attendance (1).

In agreement with other studies (1,3,4,19), we found that regular attendees were younger, mostly females, and brushed their teeth greater regularity ( Table 1). The socio-occupational profile was not statistically different, although some trends were found in the expected direction (a higher social class within the regular-attendees).

In keeping with other studies (8,9,21),our findings de-monstrate that problem-oriented attendees have poorer oral health and a poorer OHQoL than regular attendees ( Table 1). The main clinical differences were seen inthe individuals’ history of caries-related interventions, (i.e. the number of filled and missing teeth), and periodontal status (the number of healthy sextants). Nevertheless, both groups had a similar DMFT index, which can be explained in terms of the idea that routine attendees have higher likelihood of receiving restorative treatment but fewer missing teeth (see  Table 1), as reported elsewhere (22). Even after adjustment for some widely reported confounders, such as social class, age, and gender in a multivariate analysis, the problem-based attende esproved to be mostly men, with fewer healthy periodontal sextants and fewer restored but otherwise sound teeth, suffering greater pain-related impact according to the OHIP ( Table 2). Within the general Spanish population, pain-related impacts are the most prevalent (23) and the most severe (16) on the OHQoL.

The value of regular dental attendance in terms of quality of life and dental status has rarely been investigated but our results are in agreement with those studies. In one investigation involving older adults in South Australia, Ontario and North Carolina, Slade *et al*. (24) found that when dental attendance was problem- motivated it was associated with higher levels of social impact, and a poorer OHQoL. McGrath & Bedi (5) performed an epidemiological study on British Adults and found that those reporting a dental visit within the previous year felt that oral health enhanced their quality of life, after controlling for socio-demographic confounders. Recently a prospective cohort study performed on adolescents and young adults in New Zealand has shown that the long-term routine dental attendance is clearly associated with a better oral health status, as assessed both clinically and subjectively (4).

However to date, there are no data reflecting the effect of dental visiting patterns on the impact on OHQoL when patients seek dental treatment in a Public Dental Clinic. Many non-attendees may not see the need to attend a dentist until they have a problem that they consider requires attention (25,26). However, regular attendees may visit the dentist without any previously perceived impact. Thus it was expected that the impact on OHQoL of the problem-based attendees would be higher because of accumulated oral disease but also because, by definition, they only visit a dentist when oral problems arise. Nevertheless, it is also plausible that problem-based attendees would have a higher level of tolerance to oral disease (low OHQoL impact for the same oral conditions) and in this case their oral wellbeing captured by the OHIP or the OIDP should be comparable between groups. Our results demonstrate that the problem-based attendees had undergone higher pain-related impacts than their counterparts, at least in terms of the frequency of appearance (as captured by the OHIP), because in terms of severi-ty the OIDP was not a significant predictor of being a problem-based attendee ( Table 2). Future efforts should be directed towards the investigation of the level of tolerance of dental impact. The discrepancy between patients’ and clinicians’ opinions about oral health status and treatment needs has been widely discussed elsewhere by several authors (27-29), but the variable level of tolerance to oral impacts needs further research.

An alternative hypothesis for explaining our results could be proposed on the rationale that regular attendees were those with a genetically better oral health and better wellbeing, such that they are not used to receiving complex or invasive treatments because of the “healthy user” effect (30). Accordingly, they would have a lower risk of suffering from fear or anxiety when visiting a dentist, which are major reasons for refusing or delaying dental attendance (1,8,19).

We hope these preliminary results encourage the Expert Panel involved in the five-year oral health epidemiological survey among the general Spanish population to record these patterns in the next survey scheduled for 2015. To date, the only evidence available has not been published, except for internal use and has not yet reached the international arena. But it should be mentioned that according to a report available from the Spanish Dental Association website (Consejo General de Colegios de Dentistas de España) http://www.consejodentistas.es/pdf/libroblancosaludbucodnetalenespaña2010.pdf, the perception about when it is supposed to visit the dentist was estimated in a epidemiological study performed in Spain on 2010, and it was found that the percentage of subjects considering they have to visit the dentist when they have problems, increased from 12% (for young adults aged 18 to 35 years), to 16% (for adults aged 36 to 65 years) and to 28% (for elderly aged ≥66 years). All these percentages were clearly lower than that recorded on average in 1995 (40%).
